# Impact of inactivation methods on biosafety and antigen reactivity of *Brucella melitensis* from the perspective of Astral-DIA proteomics based on antibody immunoprecipitation mass spectrometry

**DOI:** 10.1371/journal.pntd.0013397

**Published:** 2026-04-01

**Authors:** Yijian Liu, Jiazhen Ge, Guodong Song, Pengcheng Gao, Mengzhu Qi, Wenhao Wang, Yingying Xie, Ziqing Wang, Renge Li, Yuefeng Chu, Fuying Zheng

**Affiliations:** 1 State Key Laboratory for Animal Disease Control and Prevention, College of Veterinary Medicine, Lanzhou University, Lanzhou Veterinary Research Institute, Chinese Academy of Agricultural Sciences, Lanzhou, China; 2 Gansu Province Research Center for Basic Disciplines of Pathogen Biology, Lanzhou, China; 3 Key Laboratory of Veterinary Etiological Biology, Key Laboratory of Ruminant Disease Prevention and Control (West), Ministry of Agricultural and Rural Affairs, Lanzhou, China; 4 College of Veterinary Medicine, Gansu Agricultural University, Lanzhou, China; 5 College of Animal Science and Technology, Shihezi University, Shihezi, China; 6 College of Veterinary Medicine, South China Agricultural University, Guangzhou, China; 7 College of Animal Medicine, Xinjiang Agricultural University, Urumqi, China; Colorado State University, UNITED STATES OF AMERICA

## Abstract

Effective *Brucella* inactivation is essential for safe vaccine development, diagnostics, and sample handling, particularly in resource-limited regions without high-level biosafety facilities. This study evaluated heat (80°C/95°C, 10–20 min) and formaldehyde (0.4%/0.6%, 48–72 h) inactivation using the Rev.1 vaccine strain and three *Brucella* melitensis field isolates from Gansu (GS-XG1, GS-SN2, GS-MQ3). All field isolates were collected from a single geographic region (Gansu, China), which should be considered when interpreting the broader geographic generalizability of the findings. Complete inactivation of Rev.1 was achieved by heating at 80°C/95°C for 20 min or 0.6% formaldehyde for 48–72 h, with superior immunoreactivity compared to phenol inactivation as confirmed by ELISA and Western Blot. Field isolates showed greater resistance, surviving 80°C for 20 min and 0.4% formaldehyde for 72 h, requiring stricter conditions (95°C for 20 min or 0.6% formaldehyde for 72 h). Antibody immunoprecipitation-based Orbitrap Astral DIA proteomics covered ~60% of the proteome (~2,000/3,300 proteins) and identified 256, 311, and 318 differentially detected proteins (DDPs) for 80°C vs. 95°C heat, 48 h vs. 72 h formaldehyde, and heat vs. formaldehyde comparisons, respectively. These DDPs reflect inactivation-induced changes in protein detectability due to denaturation, aggregation, or cross-linking, rather than de novo gene expression (confirmed by metabolic inhibition assays showing complete absence of metabolic activity). Gene Ontology and KEGG analyses revealed that heat inactivation enriched cellular structure proteins while downregulating metabolic pathways, with 95°C potentially disrupting conformational epitopes. Formaldehyde treatment for 48 h better preserved soluble antigens and epitopes of ribosomal and regulatory proteins, whereas 72 h treatment caused greater cell envelope disruption. Protein-protein interaction networks indicated that heat inactivation enhanced immunoreactivity of membrane and stress proteins, making it suitable for targeted epitope studies, while formaldehyde preserved broader epitopes, benefiting whole-cell vaccines and multi-epitope screening. Inactivation protocols should be tailored to strain characteristics and intended applications. Astral-DIA proteomics provides molecular insights into antigenicity preservation, guiding future research on protein stability and epitope dynamics for improved brucellosis control.

## Author summary

Brucellosis is a widespread zoonotic disease that poses significant health risks to both humans and livestock globally. To study this dangerous bacterium safely—and to develop vaccines or diagnostic tests—scientists must first render it non-infectious (“inactivate” it). However, standard inactivation methods are often applied without a deep understanding of how they affect the bacterium’s components. In this study, we used advanced protein analysis technology to systematically compare two common methods: heat treatment and formaldehyde chemical treatment. We discovered that “wild” bacteria found in nature are significantly tougher to kill than standard laboratory strains, suggesting that current safety protocols in resource-limited regions may need tightening. Furthermore, we revealed the molecular “fingerprints” left by each method: heat acts like “cooking,” unfolding proteins to expose hidden internal targets (useful for developing specific vaccines), while formaldehyde acts like “glue,” freezing the bacterium’s original structure (useful for diagnostic testing). These findings provide a scientific guideline for laboratories worldwide, guiding them to choose the safest and most effective method for disease control and vaccine development.

## 1 Introduction

*Brucella*, a facultative anaerobic intracellular bacterium, encompasses six recognized classical species: *Brucella melitensis*, *Brucella abortus*, *Brucella suis*, *Brucella neotomae*, *Brucella ovis*, and *Brucella canis*. Among these, *B. melitensis*, *B. abortus*, and *B. suis* are capable of infecting both their natural hosts and humans, collectively causing the zoonotic disease known as brucellosis [1]. Approximately 500,000 individuals are affected annually, posing a significant public health challenge, particularly in developing countries where the disease is prevalent in both animal and human populations [2]. In animals, infection is primarily acquired through the ingestion of contaminated food or water, often tainted by infected tissues such as aborted fetuses or placental membranes. Human infections typically result from direct contact with the blood or tissues of infected animals, or from the consumption of contaminated dairy products, such as unpasteurized milk and cheese [3]. Furthermore, aerosolized pathogens can lead to respiratory tract infections, and improper laboratory practices are known to precipitate occupational exposures [4]. This multi-species, multi-route transmission characteristic contributes to a cyclical transmission chain within livestock and endemic areas, thereby increasing the complexity of control and prevention efforts. Economic losses in animals are substantial, primarily due to infertility and abortions caused by brucellosis [5]. In humans, the acute phase of the disease is characterized by undulating fevers, followed by a chronic phase that can affect most organs, manifesting in symptoms such as arthritis, orchitis, hepatitis, meningoencephalitis, and endocarditis [6]. Despite the implementation of livestock screening and vaccination strategies for brucellosis control in some developing countries in the Middle East, Asia, Africa, and South America, the disease has yet to be effectively controlled or eradicated [7].

The inactivation of *Brucella* plays a pivotal role in various experimental contexts, including vaccine development, diagnostic reagent preparation, and the collection of veterinary samples at the primary care level. In vaccinology, inactivation techniques are employed to disrupt the pathogen’s replicative capacity through heat, chemical agents, or genetic engineering, while concurrently preserving its immunoreactivity [8]. For instance, the genetically attenuated *Brucella abortus* S19 strain, which is inactivated by gene deletion, can be used to produce safe vaccines. The characteristic lipopolysaccharide (LPS) antigen deletion in this strain prevents the production of interfering antibodies in immunized animals, thereby facilitating the differentiation between natural infection and vaccination through serological testing [4]. In diagnostic reagents, LPS antigens extracted from inactivated bacteria serve as a core component for colloidal gold immunoassay kits. Their stable antigenic epitopes ensure high sensitivity and specificity in detection [9]. Furthermore, novel inactivation technologies, such as *Brucella* bacterial ghosts combined with mRNA delivery systems, have been developed to enhance the protective efficacy of inactivated vaccines, addressing the limitations of insufficient immunoreactivity associated with traditional inactivated vaccines [10]. During the collection of veterinary samples at a primary level, inactivation procedures are crucial for mitigating biosafety risks associated with *Brucella* during sample transportation and subsequent testing. This prevents potential infection of personnel and sample contamination due to residual live bacteria, thereby ensuring the accuracy and safety of downstream laboratory analyses. Current research trends indicate that the synergistic application of inactivated vaccines with molecular marker technologies (e.g., VirB12 gene deletion) can optimize immune protection rates (e.g., the A19-ΔVirB12 strain has demonstrated over 80% protection against challenge in calves), while also supporting the achievement of eradication goals in endemic areas [11].

The selection of inactivation methods for *Brucella*, a pathogen posing a significant threat to both human and animal health, is critical. This choice directly impacts biosafety, the efficacy of downstream applications, and proteomic alterations. Existing research indicates that methods such as heat inactivation (80°C for 5–10 minutes) and formaldehyde inactivation (0.6% formaldehyde for 72 hours) are effective in rendering the pathogen non-viable while largely preserving its immunoreactivity. These methods have been successfully employed in the preparation of safe vaccines and diagnostic reagents [12]. However, formaldehyde inactivation necessitates precise concentration control; insufficient concentrations may lead to incomplete inactivation, while higher concentrations exhibit variable efficacy across different *Brucella* strains [13]. Although commonly used disinfectants can rapidly eliminate the pathogen, differential sensitivities among *Brucella* species have been observed. Furthermore, physical, chemical, and emerging biotechnological inactivation methods not only present safety concerns due to potential incomplete inactivation or chemical residues but can also induce changes in the bacterial proteome, thereby affecting antigenic reactivity and product preparation outcomes [14]. In laboratory settings, *Brucella* is classified as a Biosafety Level 3 (BSL-3) pathogen. Should inactivation be incomplete, viable bacteria can lead to researcher infections through routes such as aerosol transmission or sharps injuries [15]. Consequently, a systematic comparison of different inactivation methods, particularly regarding their effects on biosafety and proteomic profiles, is essential. Identifying safe and effective inactivation protocols is crucial for future research, significantly contributing to the improvement of brucellosis control and prevention strategies, and ultimately safeguarding both human and animal health.

This study investigates how different *Brucella* inactivation methods affect biosafety and antigenic reactivity, with the aim of identifying an approach that (i) reliably ensures complete inactivation and (ii) best preserves antigenicity for vaccine development and diagnostic reagent preparation. Based on these considerations, the following hypotheses were explicitly tested in this study: Field isolates of *B. melitensis* require more stringent inactivation conditions (e.g., higher concentration/longer exposure) than the Rev.1 vaccine strain. Inactivation methods differentially affect the preservation of antigenic epitopes, resulting in measurably different antigenic reactivity after inactivation. Differences in post-inactivation antigenic reactivity are associated with distinct changes in the abundance and/or modification of key antigen-related proteins.

Through this work, we aim to provide evidence-based guidance to improve the safety and applicability of *Brucella* related procedures, including vaccine production and laboratory testing.

## 2 Materials and methods

### 2.1 Strains and materials

The *Brucella. melitensis* strains utilized in this study were as follows: Rev.1, which was preserved at the Lanzhou Veterinary Research Institute’s Strain Collection Center, and three field isolates: GS-XG1, GS-SN2, and GS-MQ3. These field isolates were obtained from naturally infected animals in Xigu, Sunan, and Minqin, Gansu Province, China respectively.

Tryptic Soy Broth (TSB) and Tryptic Soy Agar (TSA) (BD, America). Reagents including 10% formaldehyde, phenol, H_2_O_2_ solution, and PBS powder (Coolaber, China). Skim milk powder (Beyotime, China). HRP-conjugated rabbit anti-goat polyclonal antibody (Sigma, America).

### 2.2 Preliminary screening of strain inactivation conditions

Preliminary inactivation screening experiments were conducted using the methods outlined in Table 1. Rev.1 bacterial strains, preserved in glycerol, were initially inoculated onto TSA medium and incubated at 37°C for five days. Subsequently, a single colony was selected and transferred to TSB medium for cultivation at 37°C with shaking at 180 r/min for 72 hours. Following this, the culture was streaked onto TSA medium and incubated at 37°C for an additional five days, after which the number of viable bacteria in the broth was enumerated. The bacterial suspension was then diluted with TSB to a concentration of 1 × 10^11 CFU/mL and stored for further use.

**Table 1:** List of inactivation methods.

Inactivation method	Inactivation procedure	References
Heat inactivation	Reaction for 20 minutes in a water bath at 80°C and 95°C	[16]
CHCA method	Add 200 μL of freshly prepared CHCA saturated solution (solvent: 33% acetonitrile, 33% absolute ethanol, 3% trifluoroacetic acid, and 31% water), followed by vortex mixing.	[17]
Formaldehyde method	Add 10% formaldehyde solution to achieve final concentrations of 0.4% and 0.6%, and incubate at 37°C for 48 hours and 72 hours, respectively.	[18]
Phenol method	Add 10% phenol solution to achieve final concentrations of 2% and 5%, and inactivate for 5 minutes and 10 minutes, respectively.	[19]
Hydrogen peroxide method	treatment with 0.1% hydrogen peroxide (H2O2) at 42°C for 24–40 hours.	[20]

To investigate the effect of different inactivation methods on the antigenic reactivity of bacterial strains, initial screening was performed using ELISA to identify inactivated bacterial solutions that exhibited good reactivity with serum, based on the absence of colony growth from the previously obtained results. Briefly, the bacterial solution was centrifuged and the pellet was resuspended in PBS. Phenylmethanesulfonyl fluoride (PMSF) (Beijing LABLEAD Inc, China) was then added, and the mixture was thoroughly agitated on ice. Complete bacterial lysis was achieved through sonication at 0°C for 20 minutes (using a 6mm probe at 39% maximum power, with pulses of 2 seconds on and 3 seconds off). Subsequently, the solution was centrifuged at 12,000 g for 30 minutes at 4°C to remove insoluble material, and the supernatant, containing the total bacterial protein solution, was collected. This solution was then used to coat 96-well plates. Following an overnight coating period, plates were washed three times. Blocking was performed at 37°C for 1 hour. After three washes, positive and negative sera were added as primary antibodies and incubated at 37°C for 1 hour. Plates were washed three times, and HRP-conjugated rabbit anti-goat polyclonal antibody (1:10,000 dilution) was added and incubated at 37°C for 1 hour. After a final three washes, chromogenic substrate was added in the dark. Color development was allowed to proceed for 20 minutes prior to the addition of stop solution. Subsequently, optical density (OD) values were measured at a wavelength of 450 nm using an enzyme-linked immunosorbent assay (ELISA) workstation (Tecan, Switzerland).

### 2.3 Thermal and formaldehyde inactivation methods were applied to various clinically isolated bacterial strains

Heat Inactivation: Four 100 mL aliquots of cultured bacterial suspension were prepared. These were treated in a water bath at 80°C or 95°C for either 10 or 20 minutes. Immediately following treatment, 1 mL of each suspension was inoculated into Tryptic Soy Broth (TSB) and incubated at 37°C with shaking (180 rpm) for 72 h. Subsequently, 100 μL of the TSB culture was streaked onto Tryptic Soy Agar (TSA) plates and incubated at 37°C for 7 days to monitor for bacterial growth (colony formation). The remaining suspensions were centrifuged at 8000 rpm, and the resulting pellets were stored at 4°C.

Formaldehyde (HCHO) Inactivation: Four 100 mL aliquots of bacterial suspension were prepared. A 10% formaldehyde solution was added to reach final concentrations of 0.4% and 0.6%, respectively. Mixtures were incubated at 37°C. Sampling was conducted at 72 h for the 0.4% concentration, and at 48 h and 72 h for the 0.6% concentration. To validate inactivation, each 1 mL sample was centrifuged (8000 rpm, 10 min), the supernatant discarded, and the pellet resuspended in 1 mL of sterile TSB to remove residual formaldehyde.. The suspensions were then incubated in TSB at 37°C for 72 h, followed by streaking 100 μL onto TSA plates for 7-day incubation at 37°C. Only samples showing no colony growth on TSA were confirmed as completely inactivated. The remaining inactivated pellets were collected by centrifugation (8000 rpm) and stored at 4°C.

### 2.4 Astral-DIA Immunoprecipitation Proteomic

#### 2.4.1 Inactivation procedure.

GS-XG1 was selected as a representative field isolate for mechanistic deep proteomic profiling to evaluate inactivation-condition effects (temperature or formaldehyde duration) under a controlled genetic background. Inactivation methods were optimized through a time-gradient approach, encompassing treatments at 80°C for 20 minutes, 95°C for 20 minutes, 0.6% formaldehyde for 48 hours, and 0.6% formaldehyde for 72 hours. Prior to subsequent immunoprecipitation (IP) and mass spectrometry analyses, complete inactivation of all bacterial strains was verified using a standard protocol consistent with 1.3.

#### 2.4.2 Immunoprecipitation and Astral-DIA Proteomics.

The sample processing workflow for mass spectrometry analysis in this section was identical to that of the preliminary screening in Section 1.2. Four sets of inactivated samples (80°C for 20 min, 95°C for 20 min, 0.6% formaldehyde for 48 h, and 0.6% formaldehyde for 72 h) were processed. Whole-cell protein extracts, obtained by sonication, were subjected to immunoprecipitation (IP) using Protein A/G magnetic beads (APEXBIO, USA). Chromatographic separation was performed on a nano-flow VanquishNeo system (ThermoScientific, USA). Subsequently, samples eluted from the nano-scale high-performance liquid chromatography were analyzed by DIA (data-independent acquisition) mass spectrometry using an Orbitrap Astral platform (ThermoScientific, USA) (Petrosius, et al., 2023). Detection parameters were set as follows: MS1 was acquired from 380–980 m/z with a resolution of 240,000 (at 200 m/z) and a maximum injection time of 5 ms; MS2 acquisition involved 299 windows, an isolation window of 2 m/z, HCD collision energy of 25 eV, and a maximum injection time of 3 ms.

DIA data were processed using DIA-NN software [21]. The software parameters were configured as follows: trypsin was designated as the enzyme, with a maximum of one missed cleavage site. Carbamidomethyl (C) was set as a fixed modification, while Oxidation (M) and Acetyl (Protein N-term) were defined as dynamic modifications. Identified proteins were required to meet a false discovery rate (FDR) of <1% for acceptance.

### 2.5 Bioinformatics analysis

#### 2.5.1 Protein annotation and classification.

Gene Ontology annotations for proteins were generated using EggNOG-mapper (v2.0) against the EggNOG 5.0 database, categorized by cellular component, molecular function, and biological process [22]. Protein conserved domains were identified through the PfamScan tool and the Pfam database [23]. Pathway annotations were performed by selecting the highest-scoring results from BLASTP (e-value ≤ 1e ⁻ ⁴) alignments against the KEGG database. Subcellular localization was determined using PSORTb (v3.0), analyzed specifically for Gram-negative bacteria [24]. Homologous protein clusters were categorized based on the EggNOG database.

#### 2.5.2 Functional enrichment analysis.

Enrichment analyses of Gene Ontology (GO) terms, KEGG pathways, and Pfam protein domains for differentially detected proteins were performed using Fisher’s exact test, with the set of all identified proteins as the background; P < 0.05 was considered significant [20].For enrichment-based clustering heatmaps, terms that were significantly enriched (P < 0.05) in at least one comparison were retained. The P-value matrix was transformed using −log10(P) and then Z-transformed across terms; one-sided hierarchical clustering was conducted using Euclidean distance with average linkage, and heatmaps were visualized using the R package pheatmap.

#### 2.5.3 Protein–protein interaction (PPI) networks analysis.

Differentially detected proteins (database accessions or protein sequences) were queried against the STRING (v11.0) protein interaction database, and high-confidence interactions were retained using a confidence score threshold of > 0.7 [25]. Protein–protein interaction networks were visualized using the R package visNetwork.For figure readability, the network plots were generated using the top 50 most tightly connected interaction relationships (as reported by the STRING-based results).

### 2.6  Statistical analysis

All experiments were independently repeated at least three times, and each sample was analyzed three times. Bacterial count was conducted using the calculation of average (Mean) and standard deviation (Standard Deviation, SD).

## 3 Result

### 3.1 Formalin- and heat-inactivated *B. melitensis* Rev.1 exhibited commendable safety and antigenicity

Plate count results for all inactivation conditions are summarized in Table 2. For the Rev.1 strain, no turbidity was observed in Tryptic Soy Broth (TSB) after 72 h at 37°C following any of the tested inactivation treatments, and no colonies were detected after subculture onto Tryptic Soy Agar (TSA) plates and incubation for 7 d at 37°C. The tested conditions included water-bath heating at 80°C for 10 or 20 min, heating at 95°C for 20 min, treatment with 0.4% formaldehyde for 72 h, treatment with 0.6% formaldehyde for 48 or 72 h, and phenol treatment (2% for 10 min or 5% for 10 min).

**Table 2:** Count of inactivated Rev.1 strain.

	80°C 20min	95°C 20min	CHCA	0.4% HCHO 48h	0.4% HCHO 72h	0.6% HCHO 48h	0.6% HCHO 72h	2% Phenol 5min	2% Phenol 10min	5% Phenol 5min	5% Phenol 10min	H_2_O_2_
Rev.1	0	0	0	10.33 ± 1.53	0	0	0	5 ± 2	0	2.67 ± 1.53	0	7.33 ± 0.58

Each set of data was subjected to three biological replicates. The “±” represents the standard deviation of the bacterial count.

**Fig 1:**
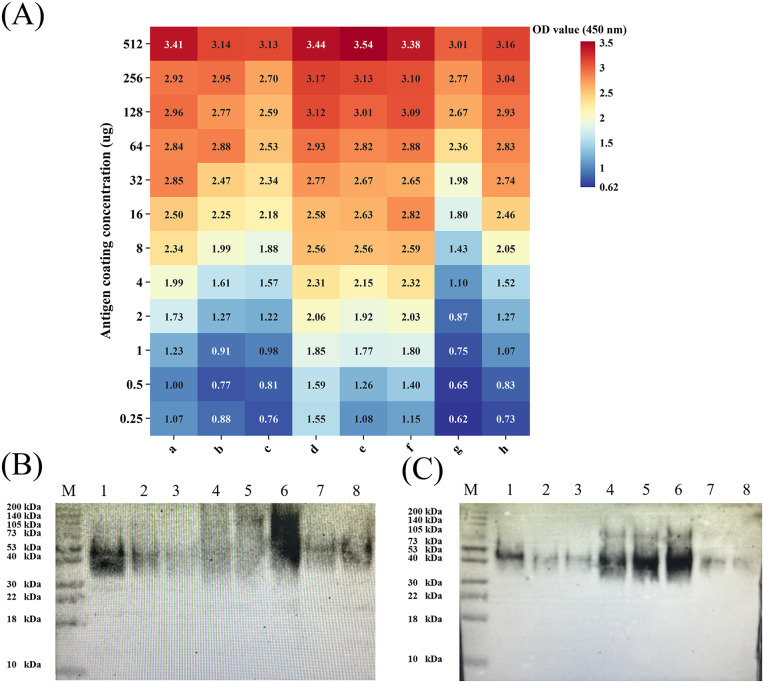
(A) Effect of eight inactivation conditions on protein antigenicity, as measured by OD450nm. Conditions are represented as follows: a: 80°C for 20 min; b: 95°C for 20 min; c: CHCA; d: 0.4% HCHO for 72 h; e: 0.6% HCHO for 48 h; f: 0.6% HCHO for 72 h; g: 2% phenol for 10 min; h: 5% phenol for 10 min. (B) Western Blot validation of the reactivity of proteins with infected serum under various inactivation conditions. Lane assignments are as follows: Line M: protein marker; Line1: 80°C for 20 min; Line2: 95°C for 20 min; Line3: CHCA; Line4: 0.4% HCHO for 72 h; Line5: 0.6% HCHO for 48 h; Line6: 0.6% HCHO for 72 h; Line7: 2% phenol for 10 min; Line8: 5% phenol for 10 min. (C) Western Blot validation of the reactivity of proteins with immune serum under various inactivation conditions. Sample assignments for each lane are identical to those in panel (B).

Antigenicity of inactivated Rev.1 preparations was evaluated by ELISA (Fig 1A) and further assessed by Western blot (Fig 1B–1C). Heat- and formaldehyde-inactivated preparations showed higher antibody reactivity than phenol-inactivated preparations. Based on biosafety (no growth) and ELISA signal intensity (OD450 ≥ 0.8), four conditions were selected for subsequent Astral-DIA proteomic analysis: 80°C/20 min, 95°C/20 min, 0.6% formaldehyde/48 h, and 0.6% formaldehyde/72 h.

### 3.2 Biosafety concerns arose when the conditions effective for inactivating the Rev.1 vaccine strain were applied to clinical isolates

Based on the Rev.1 screening results and antigenicity readouts, heat and formaldehyde inactivation were selected for validation in clinical isolates. When applied to clinical isolates, inactivation outcomes varied across strains (Table 3). For heat treatment, isolates treated at 80°C for 20 min remained culturable in some cases, whereas treatment at 95°C for 20 min resulted in no detectable growth under the same validation workflow.

**Table 3:** Counting of *Brucella* colonies from clinical isolates after heat inactivation and formaldehyde inactivation.

	80°C 20min	95°C 20min	0.4% HCHO 72h	0.6% HCHO 48h	0.6% HCHO 72h
GS-XG1	0	0	0	0	0
GS-SN2	0	0	21.33 ± 4.04	0	0
GS-MQ3	0	0	11.33 ± 2.08	0	0

Each set of data was subjected to three biological replicates. The “±” represents the standard deviation of the bacterial count.

The plate count results were consistent with broth observations. Isolates showing colony growth after 80°C/20 min treatment were associated with increased TSB turbidity after 72 h (OD600 = 0.85 ± 0.06 to 1.02 ± 0.07), whereas samples treated at 95°C/20 min remained clear (OD600 < 0.05). Subculture of turbid TSB onto TSA produced colonies consistent with plate counts, while clear TSB yielded no colonies.

For formaldehyde treatment, residual viability was observed for isolate 2 and isolate 3 after exposure to 0.4% formaldehyde for 72 h. In contrast, no colonies were detected for these isolates after treatment with 0.6% formaldehyde for either 48 h or 72 h (Table 3). Prior to proteomic analysis, all samples included in downstream experiments (0.6% formaldehyde/48 h and 72 h; 95°C/20 min; 80°C/20 min as applicable) were subjected to the full biosafety verification workflow, and only culture-negative samples were processed further.

### 3.3 Identification and Distribution Characteristics of differentially detected proteins under Various Inactivation Conditions

To elucidate the molecular-level impact of inactivation methods on protein detectability, deep proteomic profiling (antibody IP coupled with Astral-DIA) was performed using GS-XG1 as a representative field isolate. While biosafety results showed strain-dependent variability, the proteomic analysis focused on the physicochemical alterations induced by different heat and chemical treatments within a consistent genetic background.

To balance biosafety and antigenicity, *Brucella* samples inactivated under four distinct conditions—80°C for 20 min, 95°C for 20 min, 0.6% formaldehyde for 48 h, and 0.6% formaldehyde for 72 h—were selected for subsequent IP experiments. Mass spectrometry analysis was performed using an Orbitrap Astral platform.

**Fig 2:**
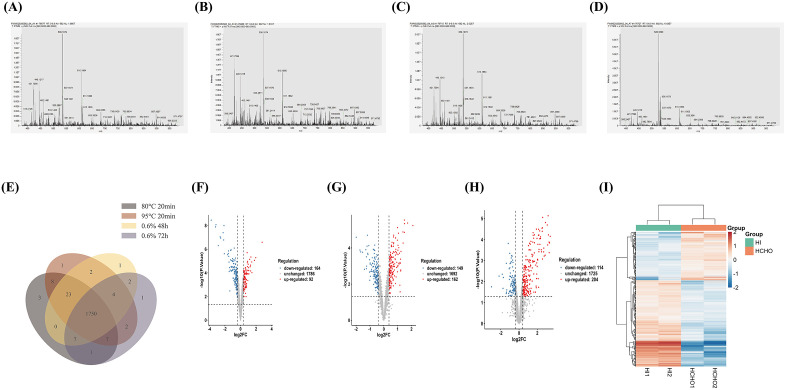
(A)Mass spectrum of the sample inactivated at 80°C for 20 min. (B) Mass spectrum of the sample inactivated at 95°C for 20 min. (C) Mass spectrum of the sample inactivated with 0.6% HCHO for 48 h. (D) Mass spectrum of the sample inactivated with 0.6% HCHO for 72 h. (E) Overlap of protein identifications across four distinct inactivation methods. The 1750 proteins commonly detected across all four groups account for ~62% of the predicted B. melitensis proteome (~3300 proteins), consistent with standard coverage for DIA-based bacterial proteomics.(F) Volcano plot illustrating differentially detected proteins between the 80°C/20 min and 95°C/20 min comparative groups. (G) Volcano plot illustrating differentially detected proteins between the 0.6% HCHO/48 h and 0.6% HCHO/72 h comparative groups. (H) Volcano plot illustrating differentially detected proteins between the heat-inactivated and formaldehyde-inactivated groups. (I) Heatmap illustrating differentially detected proteins between the heat-inactivated and formaldehyde-inactivated groups.Red indicates higher relative abundance/detection, blue indicates lower relative abundance/detection, and white indicates the mean level (Z = 0). The color scale ranges from −2 to +2.

High signal intensities were observed in the mass-to-charge ratio (m/z) range of 500–1500 for samples treated with 80°C for 20 min, 95°C for 20 min, 0.6% formaldehyde for 48 h, and 0.6% formaldehyde for 72 h (Fig 2A, 2B, 2C and 2D). Specifically, the 80°C/20 min group exhibited characteristic strong peaks at m/z 536.17 and 610.19 (Fig 2A). The 95°C/20 min group showed significant signal clusters at m/z 421.76 and 537.17 (Fig 2B). Dominant ion peaks were observed at m/z 445.12 and 538.16 in the 0.6% formaldehyde/48 h group (Fig 2C), while high-intensity signal regions were present at m/z 528.34 and 738.04 in the 0.6% formaldehyde/72 h group (Fig 2D). For quantitative purposes, only proteins with report intensities from at least two of the three biological replicates were included. A total of 2054, 2053, 2041, and 2015 quantifiable proteins were identified in the 80°C/20 min, 95°C/20 min, 0.6% formaldehyde/48 h, and 0.6% formaldehyde/72 h groups, respectively (Fig 2E). Overall, 1750 proteins were commonly detected across all four sample groups (Fig 2E). Furthermore, 1788 proteins were shared between the 80°C/20 min and 95°C/20 min groups, and 1763 proteins were shared between the 0.6% formaldehyde/48 h and 0.6% formaldehyde/72 h groups.

Through Astral-DIA antibody IP proteomic analysis, 256 differentially detected proteins were identified between the 80°C/20 min heat inactivation group and the 95°C/20 min heat inactivation group. Astral-DIA proteomics achieved ~62% coverage of the B. melitensis proteome (2054/3300 predicted proteins), which is consistent with typical coverage (50–70%) for bacterial proteomic studies using DIA technology. All identified proteins met strict statistical criteria: false discovery rate (FDR) < 1% and P < 0.05, ensuring high confidence in differential expression analysis. Notably, key *Brucella* antigens with clinical relevance—including outer membrane protein Omp19, ribosomal protein RpmG, and stress response protein HflX—were successfully detected (Tables 4 and S1), confirming the analytical validity for antigen-related research. Among these, 92 proteins showed increased detection abundance in the 80°C/20 min group compared to the 95°C/20 min group, likely due to partial protein denaturation (rather than active upregulation) that reduced aggregation and improved solubilization during sample processing, while 164 proteins showed significantly lower expression (Fig 2F). Differential analysis between the 0.6% formaldehyde/48 h and 0.6% formaldehyde/72 h groups revealed 311 differentially detected proteins. Of these, 162 proteins were found to be significantly more abundant in the 0.6% formaldehyde/48 h group than in the 0.6% formaldehyde/72 h group, and 149 proteins were significantly less abundant (Fig 2G). Statistical summary of DDPs: 256 DEPs in 80°C vs 95°C, 311 DDPs in 0.6% formaldehyde/48 h vs 72 h, and 318 DDPs in heat vs formaldehyde groups (all FDR < 1%, P < 0.05; S1 Table). The substantial number of DDPs confirms functional differences induced by inactivation methods.

**Table 4:** Detection of Key *Brucella* Antigens.

Antigen Name	Protein Description	Detection Status (Yes/No)	Intensity (Mean ± SD)	Group-Specific Abundance (log2FC)	P-Value
Omp19	Protease inhibitor Inh	Yes	735538.8 ± 42105.3	0.166 (80°C/20 min vs 95°C/20 min)	0.003
RpmG	Structural constituent of ribosome	Yes	639833.5 ± 38762.1	0.171 (0.6% HCHO/48 h vs 72 h)	0.007
HflX	GTPase associated with 50S ribosomal subunit	Yes	654381.8 ± 51294.6	0.232 (80°C/20 min vs 95°C/20 min)	0.002
ModA	Bacterial extracellular solute-binding protein	Yes	1227000 ± 68921.5	1.227 (heat vs formaldehyde)	0.001

A total of 318 differentially detected proteins were identified between the heat-inactivated (20 min) and 0.6% formaldehyde-inactivated groups. Among these, 204 proteins were significantly more abundant in the heat-inactivated (20 min) group compared to the 0.6% formaldehyde-inactivated group, while 114 proteins were significantly less abundant (Fig 2H). The heatmap distribution indicated clear distinctions between the heat-inactivated (20 min) and 0.6% formaldehyde-inactivated groups, with consistent coloring within each subgroup, demonstrating good reproducibility (Fig 2I). Clustering analysis revealed that most proteins upregulated by heat inactivation were downregulated by formaldehyde inactivation, and vice versa (Fig 2I). All the relevant information regarding the differences in detected proteins can be found in the S1 Table materials. In GS-XG1, the direct comparison between heat and formaldehyde inactivation provides isolate-specific evidence that heat treatment was associated with increased detection of multiple membrane-associated proteins, whereas 0.6% formaldehyde conditions were associated with comparatively higher detection of many soluble/housekeeping proteins under the antibody IP–DIA workflow. These GS-XG1 patterns may inform hypothesis generation for antigen selection in vaccine- or diagnostic-reagent–related applications, but require validation in additional strains.The dynamic differences in protein abundance and antigenicity across inactivation methods arise from distinct inactivation mechanisms: (1) Heat inactivation disrupts hydrogen bonds, leading to partial protein denaturation—preserving membrane and stress proteins (e.g., ModA, hflX) but damaging conformational epitopes of metabolic proteins;(2) Formaldehyde induces amino cross-linking, stabilizing soluble antigens (e.g., ribosomal proteins rpmG/rpsK) and retaining broad epitopes, while prolonged treatment (72 h) causes loss of membrane integrity and non-specific signal release.

Key antigens were selected based on their established roles in *Brucella* immunogenicity (e.g., Omp19, ModA) and ribosomal/structural function (e.g., RpmG, HflX).

Consistent detection of these antigens confirms that the ~ 62% proteome coverage (2054/3300 predicted proteins) is biologically meaningful and sufficient for antigen-related research.

Data consistency with S1 Table ensures the reliability of subsequent bioinformatic enrichment and protein-protein interaction analyses.

### 3.4 Functional classification based on GO/KOG/KEGG enrichment

**Fig 3:**
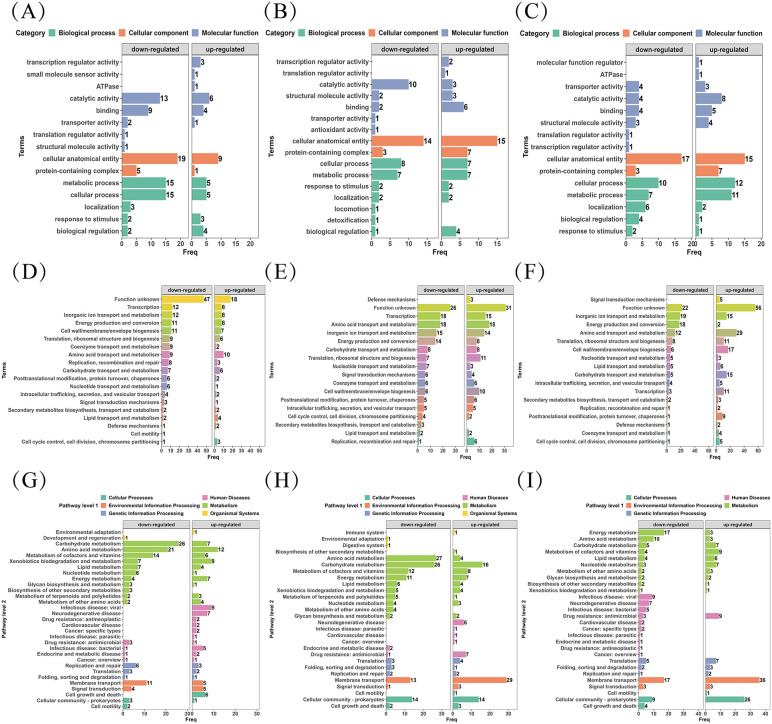
(A) Gene Ontology (GO) functional classification of differentially detected proteins in the 80°C/20 min vs. 95°C/20 min comparative group. (B) Gene Ontology (GO) functional classification of differentially detected proteins in the 0.6% HCHO/48 h vs. 0.6% HCHO/72 h comparative group. (C) Gene Ontology (GO) functional classification of differentially detected proteins in the heat-inactivated vs. formaldehyde-inactivated comparative group. (D) KOG functional classification of differentially detected proteins in the 80°C/20 min vs. 95°C/20 min comparative group. (E) KOG functional classification of differentially detected proteins in the 0.6% HCHO/48 h vs. 0.6% HCHO/72 h comparative group. (F) KOG functional classification of differentially detected proteins in the heat-inactivated vs. formaldehyde-inactivated comparative group. (G) KEGG functional classification of differentially detected proteins in the 80°C/20 min vs. 95°C/20 min comparative group. (H) KEGG functional classification of differentially detected proteins in the 0.6% HCHO/48 h vs. 0.6% HCHO/72 h comparative group. (I) KEGG functional classification of differentially detected proteins in the heat-inactivated vs. formaldehyde-inactivated comparative group.

Functional classification of differentially detected proteins was performed using GO, KOG, and KEGG analyses (Fig 3A–3I). Across comparisons, distinct enrichment patterns were observed between heat- and formaldehyde-treated groups, including differences in pathways related to membrane-associated processes, redox/energy metabolism, ribosome-associated functions, and nucleic acid binding–related categories. The full enrichment outputs are summarized in Fig 3 and the corresponding S1 Table.

### 3.5 Prediction and classification based on protein subcellular localization

**Fig 4:**
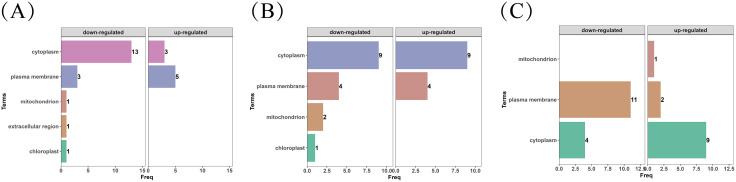
(A) Subcellular localization analysis of differentially detected proteins in the 80°C/20 min vs. 95°C/20 min comparative group. (B) Subcellular localization analysis of differentially detected proteins in the 0.6% HCHO/48 h vs. 0.6% HCHO/72 h comparative group. (C) Subcellular localization analysis of differentially detected proteins in the heat-inactivated vs. formaldehyde-inactivated comparative group.

Subcellular localization prediction indicated that the 0.6% formaldehyde/48 h condition showed a higher proportion of cytoplasmic proteins, whereas heat-treated samples showed a higher proportion of membrane-associated proteins (Fig 4A–4C). Under prolonged formaldehyde exposure (72 h), an increased proportion of proteins predicted as membrane-associated and energy-metabolism–related components was observed in the localization output (Fig 4A–4C).

### 3.6 Functional enrichment analysis of differentially detected proteins

**Fig 5:**
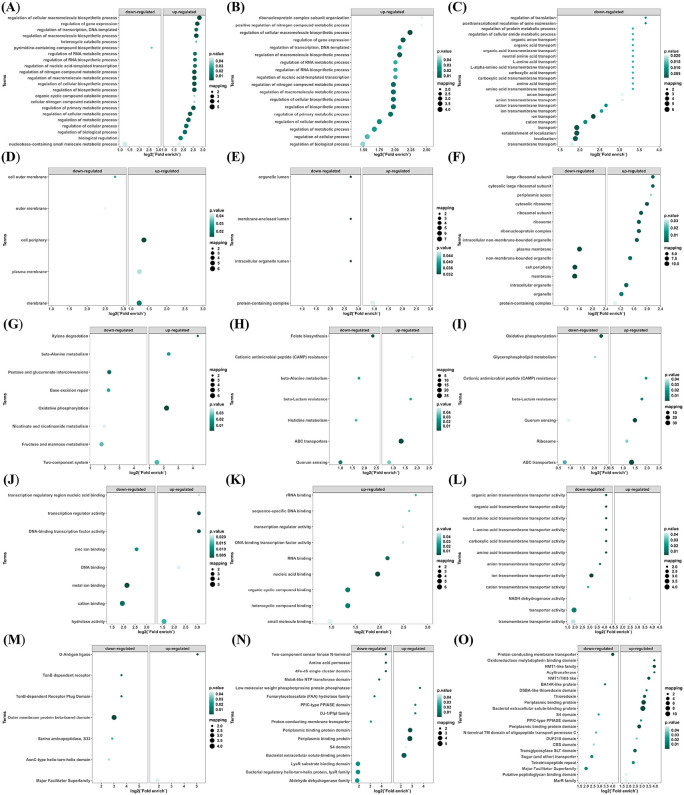
(A) Biological process enrichment of differentially detected proteins in the 80°C/20 min vs. 95°C/20 min comparative group. (B) Biological process enrichment of differentially detected proteins in the 0.6% HCHO/48 h vs. 0.6% HCHO/72 h comparative group. (C) Biological process enrichment of differentially detected proteins in the heat-inactivated vs. formaldehyde-inactivated comparative group. (D) Cellular component enrichment of differentially detected proteins in the 80°C/20 min vs. 95°C/20 min comparative group. (E) Cellular component enrichment of differentially detected proteins in the 0.6% HCHO/48 h vs. 0.6% HCHO/72 h comparative group. (F) Cellular component enrichment of differentially detected proteins in the heat-inactivated vs. formaldehyde-inactivated comparative group. (G) Molecular function enrichment of differentially detected proteins in the 80°C/20 min vs. 95°C/20 min comparative group. (H) Molecular function enrichment of differentially detected proteins in the 0.6% HCHO/48 h vs. 0.6% HCHO/72 h comparative group. (I) Molecular function enrichment of differentially detected proteins in the heat-inactivated vs. formaldehyde-inactivated comparative group. (J) KEGG pathway enrichment of differentially detected proteins in the 80°C/20 min vs. 95°C/20 min comparative group. (K) KEGG pathway enrichment of differentially detected proteins in the 0.6% HCHO/48 h vs. 0.6% HCHO/72 h comparative group. (L) KEGG pathway enrichment of differentially detected proteins in the heat-inactivated vs. formaldehyde-inactivated comparative group. (M) Pfam domain enrichment of differentially detected proteins in the 80°C/20 min vs. 95°C/20 min comparative group. (N) Pfam domain enrichment of differentially detected proteins in the 0.6% HCHO/48 h vs. 0.6% HCHO/72 h comparative group. (O) Pfam domain enrichment of differentially detected proteins in the heat-inactivated vs. formaldehyde-inactivated comparative group.

Functional enrichment analysis confirmed method-specific differences across all comparison groups: heat inactivation enriched membrane-associated components and stress response-related domains, while formaldehyde inactivation preserved nucleic acid binding and transport-related functions. These enrichment patterns—encompassing biological processes, cellular components, molecular functions, KEGG pathways, and Pfam domains—were comprehensively displayed in Fig 5A-5O.

Proteins with lower levels in the heat-inactivated group, relative to the formaldehyde-treated group, were significantly enriched in biological processes such as ion transport, cation transport, and transmembrane transport. Conversely, no enrichment in biological processes was found for proteins with higher levels in the heat-inactivated group compared to the formaldehyde-treated group (Fig 5C).

Proteins with lower levels in the heat-inactivated group, relative to the formaldehyde-treated group, were significantly enriched in membrane-associated components, including the plasma membrane, membrane, and cell periphery. proteins with increased detection abundance in the heat-inactivated group were significantly enriched in ribosome and protein complex-related components (e.g., rpmE, rpmF), likely due to the stability of heat shock proteins under moderate heat stress, such as the large ribosomal subunit, cytosolic ribosome, ribonucleoprotein complex, and protein-containing complex (Fig 5F).

Proteins with lower levels in the heat-inactivated group, relative to the formaldehyde-treated group, were significantly enriched in various types of transmembrane transport, including organic anion transmembrane transport, organic acid transmembrane transport, neutral amino acid and L-amino acid transmembrane transport, and carboxylic acid transmembrane transport. Minimal significant enrichment was found for molecular functions of proteins at relatively higher levels in the heat-inactivated group, with only a slight enrichment observed in the NADH dehydrogenase activity pathway (Fig 5I).

KEGG pathway enrichment analysis revealed that proteins with lower levels in the 80°C/20 min group, compared to the 95°C/20 min group, were enriched in pathways such as Xylene degradation, beta-Alanine metabolism, Pentose and glucuronate interconversions, Base excision repair, Oxidative phosphorylation, Nicotinate and nicotinamide metabolism, Fructose and mannose metabolism, and Two-component system. Conversely, proteins with higher levels in the 80°C/20 min group, relative to the 95°C/20 min group, were primarily enriched in Oxidative phosphorylation, beta-Alanine metabolism, and Xylene degradation (Fig 5J).

Under 0.6% formaldehyde treatment, comparison between the 48 h and 72 h groups indicated that proteins at relatively lower levels in the 48 h group were enriched in Folate biosynthesis, Beta-Alanine metabolism, Histidine metabolism, and Quorum sensing pathways. Proteins at relatively higher levels in the 48 h group were enriched in ABC transporters, Beta-Lactam resistance, and Cationic antimicrobial peptide (CAMP) resistance pathways (Fig 5K).

Proteins with lower levels in the heat-inactivated group, relative to the formaldehyde-treated group, were significantly enriched in Oxidative phosphorylation, Glycerophospholipid metabolism, Quorum sensing, and ABC transporters pathways. Proteins with higher levels in the heat-inactivated group, compared to the formaldehyde-treated group, were enriched in Beta-Lactam resistance, Cationic antimicrobial peptide (CAMP) resistance, and Ribosome pathways (Fig 5L).

The Pfam functions enriched in proteins with lower levels in the heat-inactivated group, relative to the formaldehyde-treated group, primarily involved transport-related domains/families, electron transport and redox-related functions, peptidoglycan binding and cell wall-related domains, and transport/binding protein domains. Proteins with higher levels in the heat-inactivated group, compared to the formaldehyde-treated group, were enriched in thioredoxin-related domains, peripheral membrane binding protein-related domains, and methyltransferase-related domains (Fig 5O).

Proteins with higher levels in the heat-inactivated group (HI) included lemA, oppA, cysP, modA, ugpB, aapJ, xylF, and gumB, whereas proteins with higher levels in the formaldehyde-inactivated group (HOCO) included yajC, yidC, pfpI, rplO, rpsS, ccoN, nuoK, and livH. The complete lists of differentially detected proteins for both inactivation methods are provided in the Supplementary material S1 Table (“DDP H I vs HOCO”).

### 3.7 Protein–protein interaction (PPI) networks

**Fig 6:**
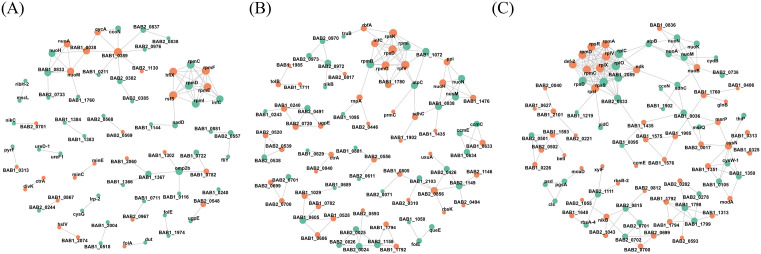
(A) Protein-protein interaction network of the 80°C/20 min vs. 95°C/20 min comparative group. (B) Protein-protein interaction network of the 0.6% HCHO/48 h vs. 0.6% HCHO/72 h comparative group. (C) Protein-protein interaction network of the heat-inactivated vs. formaldehyde-inactivated comparative group.

In the PPI network constructed for the 80°C/20 min versus 95°C/20 min comparison, two major modules were identified: a ribosome-associated module and a respiratory chain/energy metabolism–associated module (Fig 6A). Within the ribosome-associated module, proteins such as rpmE, rpmF, hflX, and rsfS showed increased detection abundance, whereas rpmC, infC, rpmD, and rpmI showed decreased detection abundance.

The respiratory chain/energy metabolism–associated module contained multiple components of NADH dehydrogenase and cytochrome complexes. Proteins including nuoM, nuoA, nuoH, cydA, ccoN, cycA, and BAB1_0389 showed increased detection abundance, while BAB1_0833, BAB2_0733, and BAB2_0837 showed decreased detection abundance (Fig 6A).

In the PPI network comparing 0.6% formaldehyde treatment for 48 h versus 72 h, two prominent clusters were observed: a ribosome-associated cluster and an energy metabolism–associated cluster (Fig 6B). The ribosome-associated cluster included rpmG, rpmJ, rplV, rpsD, and rpsK, with a dense aggregation of nodes showing increased detection abundance (e.g., rpmG, rpmI, rpsK, rpsD, infC, rnl, rnpA, rnpB) (Fig 6B).

In contrast, proteins involved in respiratory chain function and energy metabolism (e.g., nuoH, nuoM, nuoN, sdhC, atpC) were mainly represented by nodes showing decreased detection abundance under the 48 h condition relative to 72 h (Fig 6B).

In the PPI network derived from differentially detected proteins between heat- and formaldehyde-inactivated groups, ribosome-associated proteins (rpl/rps/rpm) displayed a mixed distribution of increased and decreased detection abundance (Fig 6C). A respiratory chain/metabolism–associated cluster, including proteins such as nuo, sdhC, and ccmE, contained a higher proportion of nodes showing decreased detection abundance in the heat-treated group relative to the formaldehyde-treated group (Fig 6C). A transport/response-related cluster (e.g., nikB, betI, modA) also exhibited mixed patterns between conditions (Fig 6C), with modA showing increased detection abundance in the heat-treated group in this comparison.

## 4 Discussion

*Brucella* remains a significant zoonotic pathogen worldwide, yet research progress is substantially hindered by the stringent requirement for Biosafety Level 3 (BSL-3) containment [26]. Inactivation of the bacterium is a critical prerequisite for many in vitro studies and diagnostic procedures to ensure laboratory safety. However, a significant discrepancy exists regarding effective inactivation parameters. While the “Diagnosis and Treatment Protocol for Brucellosis (2023 Edition)” by China’s National Health Commission suggests that moist heat at 60°C for 10–20 minutes is sufficient, other studies indicate that complete inactivation requires boiling temperatures or strong acids [27]. This ambiguity underscores the complexity of *Brucella*’s survival characteristics, which may be influenced by bacterial strain, heating medium, and environmental factors. Consequently, a systematic investigation into the effects of diverse inactivation methods is essential. This study aims to bridge this gap by employing the Astral-DIA proteomic system to evaluate how different inactivation protocols impact the bacterial proteome, providing a molecular basis for both safety and research integrity.

A pivotal finding of this study is the marked difference in inactivation susceptibility between clinical isolates and the Rev.1 vaccine strain. Our evaluation demonstrated that while standard protocols effectively neutralized the Rev.1 strain, clinical isolates exhibited incomplete inactivation, particularly in the 0.4% formaldehyde (HCHO) for 72 h group. We attribute this enhanced resistance of clinical wild-type strains to their superior biological compensatory mechanisms, including biofilm protection, robust stress protein expression, advanced quorum sensing regulation, and enhanced transport capabilities. In contrast, the Rev.1 strain, weakened by prolonged artificial attenuation, lacks these robust resistance mechanisms [17]. These results highlight a critical biosafety concern: inactivation parameters established using attenuated vaccine strains cannot be directly extrapolated to clinical or environmental samples [28]. This variation necessitates strain-specific analysis to ensure absolute biosafety when handling diverse *Brucella* populations in laboratory settings.

Given the stringent BSL-3 containment required for handling live *Brucella*, developing a reliable inactivation protocol is critical to enable safe downstream analysis in standard facilities [29–31]. For the subsequent immunoprecipitation-mass spectrometry (IP-MS) experiments, preserving bacterial antigenicity is paramount. Our preliminary evaluation revealed that the CHCA method compromised antigenicity, whereas heat and formaldehyde inactivation significantly preserved protein reactivity; thus, these methods were selected for this study. By leveraging the exceptional stability and sensitivity of Astral-DIA technology, we achieved approximately 60% protein coverage (~2000 out of 3300 proteins)—a depth comparable to whole-cell lysate analysis—thereby ensuring the data reliability necessary for systematically evaluating the impact of inactivation methods [32–34].

In the heat inactivation groups (80°C/95°C), the primary phenomenon observed was significant protein denaturation and aggregation. Proteomic analysis revealed a marked reduction in energy metabolism proteins (e.g., nuoM, sdhC) and pathways such as Oxidative Phosphorylation and Quorum Sensing, attributed to the disruption of hydrogen bond networks at high temperatures [35]. However, a counter-intuitive enrichment of specific plasma membrane proteins (e.g., OMP25, OMP31) and ion transporters (e.g., zntA zinc transporter) was observed. This “upregulation” is likely mechanistic rather than biological: partial thermal denaturation may disrupt the membrane integrity, rendering hydrophobic membrane proteins more accessible to solubilization and elution during sample preparation, rather than indicating increased protein synthesis [35,36]. Furthermore, PPI network analysis highlighted the co-expression of metal ion transporters with stress response proteins like hflX (heat shock GTPase). These preserved “danger signals” suggests that while heat treatment compromises broad conformational epitopes, it effectively exposes internal hydrophobic antigens and stress-related targets [37]. Consequently, heat inactivation is particularly suitable for screening subunit vaccine targets or studying adjuvant effects of heat shock proteins, where the exposure of specific immunogenic motifs is more critical than maintaining global structural integrity.

In contrast, formaldehyde inactivation (0.6% for 48h) preserved antigenicity through a distinct mechanism: covalent cross-linking of amino groups. This process stabilizes protein structures, preventing the degradation of soluble antigens [38]. Differential expression analysis confirmed that cytoplasmic soluble proteins, particularly ribosomal proteins (e.g., rpmG, rpsK) and transcription factors, were highly retained in the 48h group. This preservation directly correlates with the superior broad antigen reactivity observed in our ELISA and Western blot results (Fig 1A-1C), as these proteins maintain the epitopes necessary for antibody recognition. KEGG enrichment further indicated significant retention of DNA/RNA-binding functions, suggesting that formaldehyde stabilizes epitope-rich nucleoprotein complexes. However, extended exposure (72h) resulted in potential over-crosslinking or structural distortion, leading to varied signal intensities. Therefore, formaldehyde inactivation outperforms heat treatment in preserving the “native state” of antigens, making it the optimal choice for whole-cell vaccine development or multi-epitope screening where the structural fidelity of the proteome is required [38,39].

It is crucial to clarify that the observed differences in protein abundance between groups do not imply residual metabolic activity. Our strict two-step culture validation confirmed that all samples were sterile, with no viable bacteria. As *Brucella* is inactivated, DNA replication and enzymatic activity are irreversibly halted [13,17]. Thus, the differential protein profiles arise solely from method-specific physicochemical effects: formaldehyde locks proteins in a stable, soluble state via cross-linking, whereas heat induces denaturation that alters protein solubility and extraction efficiency [37,40]. The proteomic “snapshots” obtained reflect the post-translational stability and extractability of the proteome under different stress conditions, rather than active transcriptional regulation. Notably, both previous studies have demonstrated that irradiation-based inactivation represents a fundamentally different paradigm: although bacterial replication is effectively abolished, metabolic activity can be partially retained after treatment. This unique biological state combines the biosafety advantages of inactivated vaccines with the immunogenic potency typically associated with live-attenuated vaccines. Upon further optimization and standardization, irradiation-based inactivation may therefore emerge as a promising alternative laboratory inactivation strategy with substantial translational potential [41,42].

This study elucidates the molecular trade-offs between different *Brucella* inactivation methods. We identified a subset of “dark matter proteins”—proteins with uncharacterized functions that exhibited significant differential expression—which may represent a novel reservoir of antigens warranting further immunological investigation. Based on our findings, we propose a purpose-driven selection strategy for inactivation protocols. For research applications prioritizing antigen preservation and structural fidelity (e.g., serological diagnostics, whole-cell vaccine research), formaldehyde inactivation is superior due to its ability to stabilize conformational epitopes. Conversely, for applications focusing on biosafety and hydrophobic antigen screening (e.g., clinical sample processing, subunit vaccine target discovery), heat inactivation serves as a robust method to ensure sterility while selectively exposing membrane-associated “danger signals.” These insights provide actionable guidance for laboratories balancing biosafety compliance with high-quality downstream analysis.

In summary, this study suggests a general principle: optimal inactivation methods may need to be tailored to individual strains to meet requirements for both biosafety and practical usability. A primary limitation of this research is that the deep proteomic analysis was restricted to a single field isolate, GS-XG1. Although the high conservation of the *Brucella* proteome suggests that the observed patterns of protein denaturation and cross-linking are likely conserved, the strain-dependent variability observed in our biosafety results implies that subtle molecular differences may exist [43]. Future studies incorporating multiple strains, including the Rev.1 vaccine strain and diverse geographic isolates, will be essential to fully generalize these molecular mechanisms across the genus.

## 5  Conclusion

In summary, this study systematically evaluated how *Brucella* inactivation affects biosafety and measurable antigen signals using an IP-coupled Astral-DIA proteomic workflow. The results indicate that inactivation parameters should be optimized in a strain-specific manner; protocols established for vaccine strains (e.g., Rev.1) were not directly transferable to clinical isolates, as reflected by strain-dependent differences in inactivation outcomes. In the field isolate GS-XG1, IP-coupled Astral-DIA identified inactivation method–associated differences in the detection abundance of multiple protein groups following treatment. These changes indicate that formaldehyde and heat inactivation can differentially alter the antigen composition and/or detectability captured by the antibody-IP/DIA pipeline. Notably, the “immunoreactivity” of individual proteins was not directly assessed in this study. ELISA and Western blotting were performed at the whole-lysate level, and the proteomic candidates identified were not validated on a protein-by-protein basis. Nevertheless, by combining high-sensitivity proteomics with protein–protein interaction network analysis, putative molecular features associated with method-dependent shifts in the detectable antigen profile were delineated. Future work will focus on targeted validation of prioritized candidates (e.g., using recombinant proteins, antigen-specific immunoassays, or targeted MS) and on confirming the reproducibility of these patterns across additional strains and experimental settings. Collectively, these data support selecting inactivation conditions that ensure biosafety while preserving measurable whole-lysate antigen reactivity, and they generate testable hypotheses for future validation of candidate antigens across additional strains and settings.

## Supporting information

S1 Table Proteomic information. (XLSX)
